# Rapid Identification and Isolation of Inhibitors of Rat Lens Aldose Reductase and Antioxidant in* Maackia amurensis*

**DOI:** 10.1155/2017/4941825

**Published:** 2017-04-06

**Authors:** Set Byeol Kim, Seung Hwan Hwang, Zhiqiang Wang, Jae Myung Yu, Soon Sung Lim

**Affiliations:** ^1^Department of Food Science and Nutrition, Hallym University, 1 Hallymdeahak-gil, Chuncheon 24252, Republic of Korea; ^2^Hallym University Kangnam Sacred Heart Hospital, 1, Singil-ro, Yeoungdeungpo-gu, Seoul 07441, Republic of Korea; ^3^Institute of Natural Medicine, Hallym University, 1 Hallymdeahak-gil, Chuncheon 24252, Republic of Korea

## Abstract

Oxidative stress and aldose reductase activity have been implicated in the development of diabetic complications. In this study, the antioxidant and aldose reductase (AR) inhibitory effects of* Maackia amurensis* (MA) were investigated. The ethyl acetate fraction of the MA extract showed the highest inhibitory activity in antioxidant and rat lens AR (RLAR). To identify and isolate the active components in the ethyl acetate fraction of the MA extract, high-speed countercurrent chromatography and Sephadex LH-20 column chromatography were performed and guided by an offline HPLC-ABTS assay and HPLC microfractionation AR assay. Four antioxidants, namely, piceatannol (IC_50_ = 6.73 *μ*M), resveratrol (IC_50_ = 11.05 *μ*M),* trans*-ferulic acid (IC_50_ = 13.51 *μ*M), and chlorogenic acid (IC_50_ = 27.23 *μ*M), and six AR inhibitors, namely, chlorogenic acid (IC_50_ = 4.2 *μ*M), tectoridin (IC_50_ = 50.4 *μ*M), genistein (IC_50_ = 57.1 *μ*M), formononetin (IC_50_ = 69.2 *μ*M), resveratrol (IC_50_ = 117.6 *μ*M), and daidzein (IC_50_ = 151.9 *μ*M), were isolated and identified. The screening results of the offline HPLC-ABTS assay and HPLC microfractionation AR assay matched the activity of isolated compounds. Thus, MA is potentially valuable for antioxidant and AR inhibitor discovery and efficient drug design for the prevention and treatment of diabetic complications.

## 1. Introduction

Hyperglycemia in diabetes mellitus is considered the primary factor for the pathogenesis of long-term diabetic complications, including retinopathy, cataractogenesis, nephropathy, and neuropathy. The polyol pathway is a particularly important mechanism in diabetic complications [1, 2]. Aldose reductase (AR, EC.1.1.1.21) is the key rate-limiting enzyme in the polyol pathway and catalyzes the reduction of glucose, in the presence of NADPH, to sorbitol, which can be further oxidized to fructose by sorbitol dehydrogenase in the presence of NADH. During this process, the rate of glucose reduction to sorbitol is faster than that of sorbitol oxidation to fructose. Intracellular accumulation of the osmotically active sorbitol gives rise to osmotic stress and swelling, and thus the membrane permeability changes, promoting cataractogenesis in the lens [3]. Moreover, the changes in the NADPH/NADP^+^ and NADH/NAD^+^ ratios may induce redox imbalance and oxidative stress, further damaging tissues in patients with diabetes [4]. Also, the produced fructose from the polyol pathway is an important contributor to the formation of advanced glycation end products, which cause the dysfunction of vascular wall components [5]. Thus, development of antioxidants and inhibitors of AR to ameliorate oxidative stress and prevent the polyol pathway, respectively, is important for the treatment of diabetic complications. Currently, many potent and active synthetic agents, such as aminoguanidine, metformin, carnosine, and tenilsetam, have been developed as AR inhibitors and antioxidants; however, because of concerns about their adverse effects, there is increasing interest in the development of new AR inhibitors and antioxidants from natural sources [6].

*Maackia amurensis* (MA) is a deciduous tree distributed widely in the northeast of China as well as in the southern part of the Russian Far East and North Korea. The dried stem bark of this plant has been used as folk medicine for the treatment of cancer, cholecystitis, and arthritis [7]. In previous reports, MA has been found to contain various flavones, isoflavones, stilbenes, pterocarpans, dimeric stilbenes, and so forth [8, 9].

To identify and isolate bioactive components from natural products, bioassay-guided fractionation is commonly used; however, this old-fashioned approach is time-consuming and labor intensive [10]. Since the advent of high-throughput screening in the early 1990s, offline HPLC-based activity profiling has been proposed and implemented for the effective tracking of bioactive compounds in natural product extracts. Consequently, in recent times, many offline HPLC-based assays have been developed, and many studies have reported successful application of target isolation, that is, offline HPLC-ABTS assay and microfractionation bioassays [11].

To date, however, no data are available on the inhibitory effects of the MA and its constituents on AR. Therefore, as part of our continuing search for new AR inhibitors and antioxidants from natural products, we were to investigate the antioxidant and AR inhibitory effects of the dried stem bark of MA and isolate its active components using high-speed countercurrent chromatography (HSCCC) and Sephadex LH-20 column chromatography guided by an offline HPLC-ABTS assay and HPLC microfractionation AR assay.

## 2. Materials and Methods

### 2.1. General Points

^1^H and ^13^C NMR spectra and correlation 2D NMR spectra were obtained from a Bruker Avance DPX 400 (or 600) spectrometer. These spectra were obtained at operating frequencies of 400 MHz (^1^H) and 100 (or 150) MHz (^13^C) with CD_3_OD, (CD_3_)_2_SO, (CD_3_)_2_CO, or D_2_O and TMS used as an internal standard; chemical shifts were reported in *δ* values. The molecular mass was measured using the Voyager DE STR matrix assisted laser desorption/ionization time-of-flight (MALDI-TOF) mass spectrometer (MS, Applied Biosystems, Foster City, CA, USA), the low resolution-electronic impact (EI) MS equipped JMS-700 (Tokyo, Japan). Fast atom bombardment (FAB) MS was recorded in the negative form using* m*-nitrobenzyl alcohol as matrix in a JEOL JMSAX 505-WA spectrometer (Tokyo, Japan).

### 2.2. Reagents and Materials

Nicotinamide adenine dinucleotide phosphate (NADPH), DL-glyceraldehyde dimer, 2,2′-azino-bis(3-ethylbenzothiazline-6-sulfonic acid) diammonium salt (ABTS), 6-hydroxy-2,5,7,8-tetrame-thylchroman-2-carboxylic acid (Trolox), aminoguanidine, and quercetin were purchased from Sigma-Aldrich (St. Louis, MO, USA). Ultrapure water used for all solutions was obtained using a Milli-Q laboratory water purification system (Millipore, Bedford, MA, USA) with a resistivity over 18.2 MΩ cm. All solvents used were purchased from J. T. Baker (Phillipsburg, NJ, USA) and reagents used were purchased from Sigma-Aldrich Co., unless stated otherwise. Plant material (MA, voucher number RIC-2015-7) used in this study was purchased from a local market in Chuncheon, Gangwondo, South Korea.

### 2.3. Preparation of Extract Sample

The dried bark of MA (1.2 kg) was refluxed twice with 70% ethanol extract for 3 h each. The solvent was evaporated under reduced pressure at 40°C to give a 70% ethanol extract (yield: 7.87%). This extract was suspended in distilled water (H_2_O) and then successively partitioned with* n*-hexane (*n*-Hex), methylene chloride (CH_2_Cl_2_), ethyl acetate (EtOAc),* n*-butanol (*n*-BuOH), and H_2_O to yield the* n*-Hex fraction (0.60 g), CH_2_Cl_2_ fraction (5.05 g), EtOAc fraction (14.76 g), and* n*-BuOH fraction (9.85 g), as well as the H_2_O fraction (11.05 g). Each extract was dried by rotary evaporation at 40°C, while the H_2_O fraction was freeze-dried. The EtOAc fraction showed strong inhibitory activities on RLAR and ABTS. Therefore, this fraction was used for ABTS-offline HPLC analysis, HPLC microfractionation AR assay, and isolation.

### 2.4. HPLC Analysis

HPLC equipment was an Agilent 1200 series instrument (Agilent Technologies, Seoul, Korea) consisting of a vacuum degasser (G1322A), a quaternary pump (G1311A), an autosampler (G1329A), a thermostatted column compartment (TCC, G1316A), and a variable wavelength detector (VWD, G1314D) system. HPLC were achieved using a Gemini column (150 × 4.6 mm i.d., 5 *μ*m particle size; Phenomenex). The mobile phase, consisting of 0.1% aqueous trifluoroacetic acid and acetonitrile, was used at a flow rate of 0.7 mL min^−1^. The gradient elution program was modified as follows for a total of 70 min: 0–20% B (0–10 min), 20-20% B (10–15 min), 20–25% B (15–25 min), 25–30% B (25–35 min), 30-30% B (35–40 min), and 30–100% B (40–50 min). Injection volume was 10 *μ*L at a sample concentration 1 mg mL^−1^, and the detection wavelength was 254 nm.

### 2.5. Isolation and Identification of Activity Compounds

#### 2.5.1. Distribution of Two-Phase Solvent System

For peaks divided into upper and lower layers, the solvent fraction had the preference with n-hexane-ethyl acetate-methanol-water (2 : 8 : 1 : 9, v/v). Then, the upper layer was isolated by HSCCC and the lower layer was insulated using Sephadex LH-20.

#### 2.5.2. Preparation of Two-Phase Solvent System and Sample Solution

The two-phase solvent systems were tested to select a suitable solvent system based on the partition coefficient (*K*). Ten milligrams of the EtOAc fraction from the MA extract was weighed in a 20 mL test tube and 5 mL of each phase was added, which preequilibrated a two-phase solvent at room temperature. After the tube was strongly shaken, the solution was checked for a settling time that is closely correlated to the retention of the stationary phase, and then each phase was analyzed by HPLC to obtain the *K* value of the target compound. The *K* value was calculated as the peak area in the upper phase divided by the lower layer and then the upper phase was used as the stationary phase, and the lower phase was used as the mobile phase.

#### 2.5.3. High-Speed Countercurrent Chromatography (HSCCC)

The HSCCC instrument was a model TBE-1000A HSCCC (Tauto Biotechnique Company, Shanghai, China) with three multilayer coil columns (*ID* of the tubing: 1.8 mm, column volume: 260 mL) connected in series and a 50 mL sample loop. The *β* value (*β* = *r*/*R*, where *r* is the distance from the coil to the holder shaft and *R* is the distance between the holder axis and central axis of the centrifuge) of the multilayer coil varies from 0.60 (internal terminal) to 0.80 (external terminal). The revolution speed of the apparatus was regulated at 0–1000 rpm with an electronic speed controller. The HSCCC system was equipped with a Model Hitachi L-6200 intelligent pump (Hitachi, Tokyo, Japan) and an Isolera FLASH purification system (Biotage, Uppsala, Sweden) as UV monitor. The multilayer coil column was first entirely filled with the upper organic phase at a flow rate of 20 mL min^−1^. The lower aqueous phase was pumped into the inlet column as the mobile phase at 5 mL min^−1^, while the apparatus was rotated at 400 rpm. The mode for HSCCC separation was “head to tail.” After the hydrodynamic equilibrium was established, the EtOAc fraction of the MA extract (2 g in 40 mL of each phase) was injected into the separation column through the injection valve, and then each peak fraction was collected in 25 mL tubes while monitored with a UV detector at 254 nm.

#### 2.5.4. Sephadex LH-20 Column Chromatography

A glass column (90 cm × 3 cm i.d.) was packed with Sephadex LH-20 gel in 60% methanol at room temperature. Then, 0.49 g of the EtOAc fraction of the MA extract in 1.5 mL of 60% methanol was loaded to the column and eluted.

### 2.6. Evaluation of Trolox Equivalent Antioxidant Capacity (TEAC)

ABTS radical scavenging activity was evaluated by modifying a previously described protocol [12, 13]. A 2 mM ABTS stock solution was mixed with 3.5 mM potassium persulfate in distilled water in a bottle wrapped with foil and stored at room temperature for 12 h until the reaction was complete and the absorbance was stable. To determine scavenging activity, 10 *μ*L of sample (3 mg mL^−1^ in DMSO) was mixed with 290 *μ*L ABTS solution in a 96-well microplate and incubated at room temperature for 10 min. Then, the mixture was measured at 750 nm using a microplate reader and DMSO as a control. Trolox was used as a positive control. The TEAC results are calculated as IC_50_ values (standard error mean of triplicate experiments) as well as Trolox equivalents (TEAC).

### 2.7. ABTS-Offline HPLC Assay Analysis

In total, 10 *μ*L of the EtOAc fraction from the MA extracts (20 mg mL^−1^ in methanol) was mixed with 140 *μ*L ABTS solution that was prepared one day before. Then, the mixture was incubated at room temperature for 10 min, and then the mixture was filtered through a 0.45 *μ*m filter to HPLC analysis. The EtOAc fraction of the MA extract (20 mg mL^−1^ in methanol) was used as a control. The extents of peak decrease are expressed as a quantitative reduction.

### 2.8. Assay for Rat Lens AR Inhibitory Activity

Rat lens (RL) homogenate was prepared according to the modified method of Hayman and Kinoshita [14, 15]. RL were removed from the eyes of male Sprague-Dawley rats weighting 250–280 g and were frozen until use. RL were homogenized in 0.10 M sodium phosphate buffer (pH 6.2), which was prepared the previous day in sodium phosphate dibasic (Na_2_HPO_4_·H_2_O, 0.66 g) and sodium phosphate monobasic (NaH_2_PO_4_·2H_2_O, 1.27 g) in 100 mL of distilled water. The supernatant was obtained by centrifugation of the homogenate at 10,000 rpm at 4°C for 20 min and was frozen until use. A partially purified enzyme with a specific activity of 6.5 U mg^−1^ was routinely used to test enzyme inhibition. Each 1 mL cuvette contained 531 *μ*L of 100 mM sodium phosphate buffer (pH 6.2), 90 *μ*L of AR homogenate, 90 *μ*L of 1.6 mM NADPH, 9 *μ*L of the samples dissolved in DMSO, and 90 *μ*L of 25 mM of DL-glyceraldehyde as the substrate. The RLAR activity was assayed spectrophotometrically by measuring the decrease in the absorption of NADPH at 340 nm over a 4 min period [2].

### 2.9. HPLC Microfractionation of Rat Lens AR

An automated fraction collector (Foxy 200, ISCO, Lincoln, NE, USA) connected to HPLC equipment was used to separate and collect compounds from extracts directly into 96-well plates (Nunc, Roskilde, Denmark), with 0.35 mL in each well. After collection, 96-well plates were evaporated to dryness using an EZ-2 plus evaporator (Genevac Ltd., Ipswich, UK), and RLAR inhibitory activity was evaluated as described in upper section.

### 2.10. Statistical Analysis

All data was expressed as mean ± SD values from triplicates, analyzed via a one-way analysis of variance (ANOVA) with significant difference between means determined at *p* < 0.05, and measured with Duncan's multiple range tests using the Statistical Package for Social Science Research version 19 (SPSS).

## 3. Results

### 3.1. Determination of Active Target Fraction from the Five Fractionation Extracts of* Maackia amurensis*

The ethanol extract of MA was fractionated by increasing polarity solvents and was then measured. Antioxidant activity was measured by evaluating the decrease of absorbance at 750 nm in TEAC assay. The EtOAc fraction had superior activity in ABTS assays with IC_50_ values of 4.92 *μ*g mL^−1^ and a TEAC value of 1.77 as compared to positive control (Trolox, IC_50_ = 8.72 *μ*g mL^−1^; TEAC 1.00) (Table 1). The IC_50_ values of RLAR inhibition, except for the EtOAc fraction from the 70% ethanol extract, were higher than 100 *μ*g mL^−1^. The EtOAc fraction showed potent inhibition of RLAR with IC_50_ value of 11.3 *μ*g mL^−1^, as compared to the positive control (quercetin), a well-known AR inhibitor, with an IC_50_ value of 3.3 *μ*g mL^−1^ (Table 1).

### 3.2. ABTS^+^ Offline HPLC Radical Scavenging Analysis

**Figure 1:**
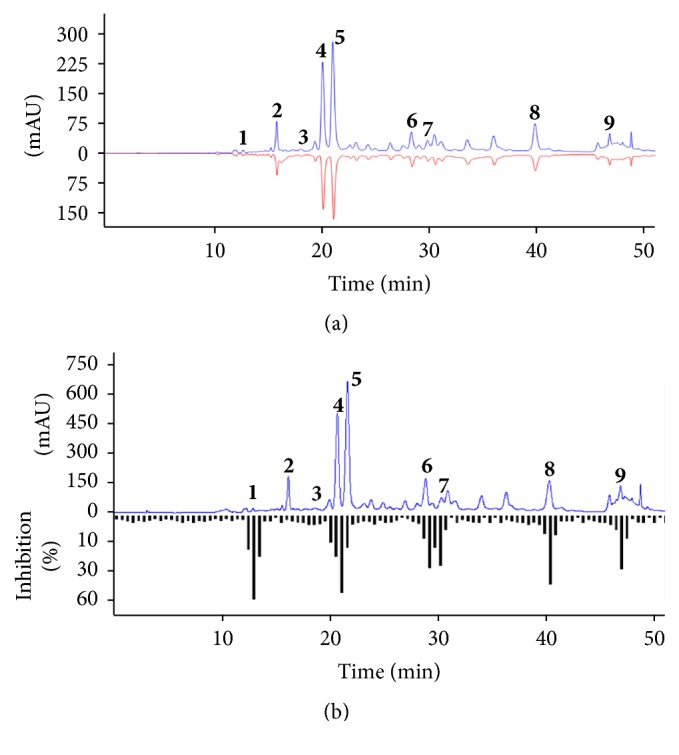
Screening test from the ethyl acetate fraction of* Maackia amurensis* by offline HPLC. The peak before reaction is the upper line and the peak after reaction is the inverse line in each chromatography. (a) The result of ABTS-offline HPLC for antioxidants. (b) The result of HPLC microfractionation for RLAR inhibition.

In order to identify the antioxidants in the EtOAc fraction of the MA extract, an offline HPLC-ABTS assay was developed. The EtOAc fraction of the MA extract spiked with ABTS solution was measured at 254 nm. Accordingly, as shown in Figure 1(a), peaks 1, 3, 5, and 6 showed peak area reductions, while peak areas of 2, 4, and 7–9 were slightly decreased. Therefore, peaks 1, 3, 5, and 6 were considered as potential antioxidants based on the relative peak areas in the HPLC chromatogram.

### 3.3. Rat Lens AR Inhibition Screening for Using HPLC Microfractionation

According to our results it is necessary to confirm the presence of effective RLAR inhibitory compounds in the EtOAc fraction of the MA extract. The EtOAc fraction of the MA extract (20 mg) was microfractioned by HPLC and the fractions were collected in a 96-well plate, of which the RLAR inhibitory activity was then assessed. As shown in Figure 1(b), the RLAR inhibitory activity in the corresponding wells of peak 1, peak 4, peak 6, peak 7, peak 8, and peak 9 was 43.2%, 41.89%, 43.31%, 35.14%, 45.54%, and 44.11%, respectively.

### 3.4. Isolation of Active Target Peaks by HSCCC and Sephadex LH-20

#### 3.4.1. Selection of Two-Phase Solvent System and Other Conditions of HSCCC

**Figure 2:**
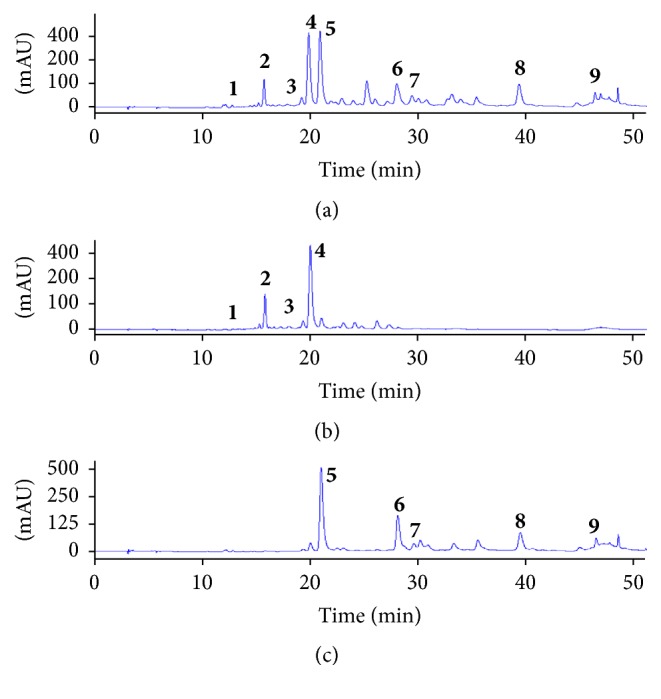
HPLC analysis of ethyl acetate fractions from* Maackia amurensis* by an* n*-hexane-EtOAc-methanol-water (2 : 8 : 1 : 9, v/v) solvent system divided into two layers. (a) EtOAc fraction of* Maackia amurensis*. (b) The lower layer is included in methanol-water solution. (c) The upper layer is included in* n*-hexane-EtOAc solution.

**Figure 3:**
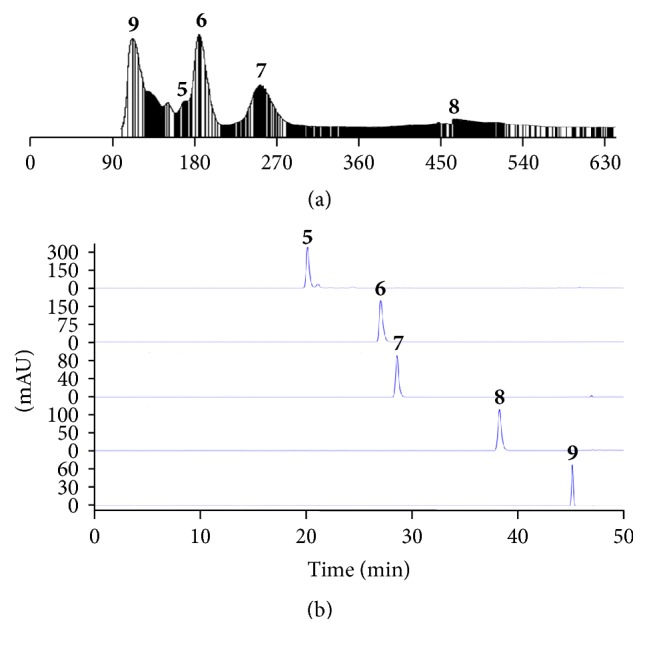
HSCCC separation of active compounds from an ethyl acetate fraction of* Maackia amurensis*. (a) The upper layer from an ethyl acetate fraction of* Maackia amurensis* from an HSCCC separation solvent system:* n*-hexane-EtOAc-methanol-water (2.5 : 7.5 : 5 : 5, v/v); flow rate, 4.0 mL/min; revolution speed, 500 rpm; sample size, 2.0 g; injection volume, 40 mL; detection wavelength, 254 nm. (b) HPLC analysis of isolated compounds by an HSCCC system; peak 5, piceatannol; peak 6, resveratrol; peak 7, daidzein; peak 8, genistein; peak 9, formononetin.

In order to confirm the inhibitory activities of potent inhibitors, polarity was divided into two layers for easy separation, and then HSCCC and Sephadex chromatography were performed for isolation of each target. In this study, the *K* value of the EtOAc fraction of the MA extraction compounds was calculated by HPLC. Before HSCCC separation, the EtOAc fraction of MA extract was fractionated by the solvent fraction with n-hexane-ethyl acetate-methanol-water (2 : 8 : 1 : 9, v/v) to separate active compounds. The EtOAc fraction was divided into two layers (Figure 2), and then five peak values in the upper layer from the EtOAc fraction of the MA extract were determined, as described in Materials and Methods (Table 2). Considering the above three factors, one system was chosen, with n-hexane-ethyl acetate-methanol-water (2.5 : 7.5 : 5 : 5, v/v) suitable for all five peaks. As shown in Figure 3, we isolated five compounds (5–9) and the isolated compounds evaporated to yield 170.1, 94.6, 8.2, 10.4, and 5.5 mg at 89.7, 91.2, 95.3, 86.8, and 93.1% purity, respectively, as determined by HPLC.

#### 3.4.2. Isolation of Compounds 1–4 by Using Sephadex LH-20

**Figure 4:**
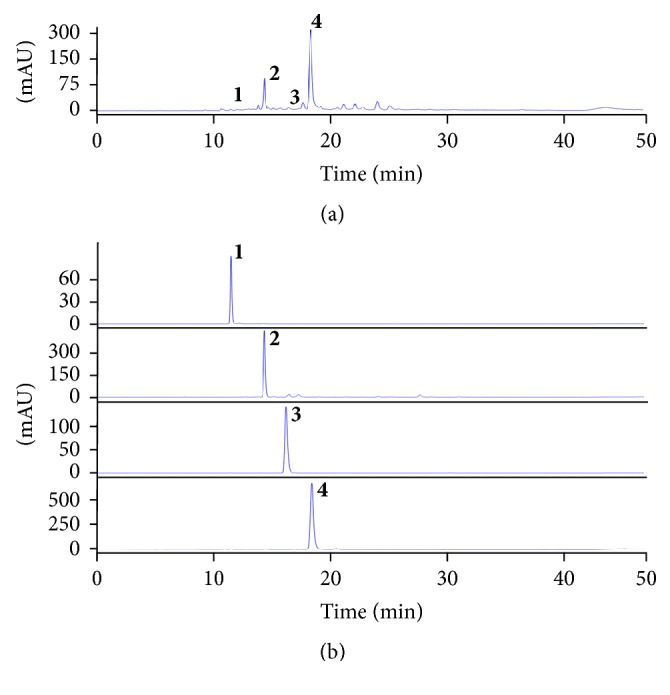
The lower layer of ethyl acetate soluble fraction from* Maackia amurensis* was isolated with Sephadex LH-20 and isolated compounds were monitored by HPLC analysis at 254 nm. (a) The lower layer of EtOAc fraction from* M. amurensis*. (b) Isolated compounds; peak 1, chlorogenic acid; peak 2, formononetin-7-*O*-*β*-D-glucosyl [1–6] glucoside; peak 3, trans-ferulic acid; peak 4, tectoridin.

**Figure 5:**
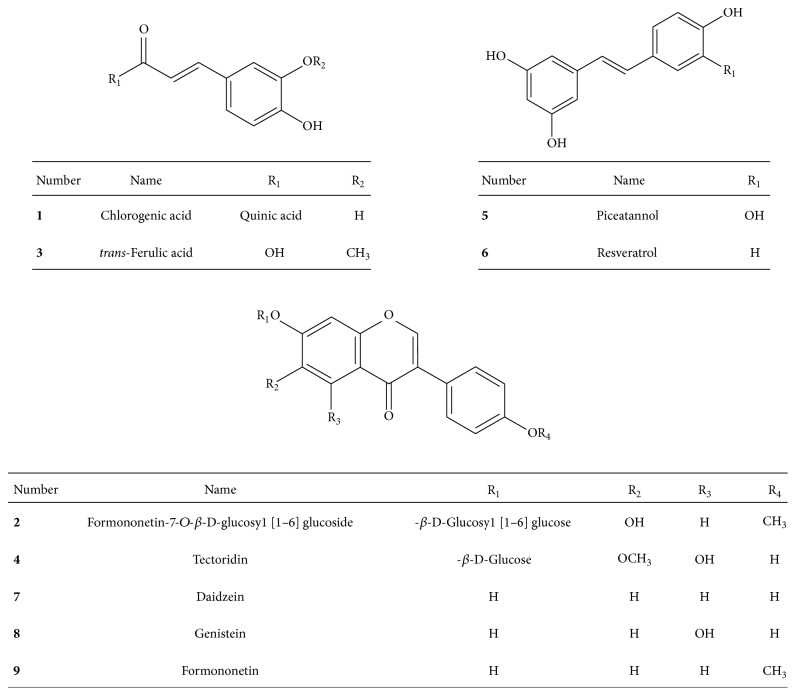
Chemical structure of compounds identified from the ethyl acetate fraction of* Maackia amurensis*.

The lower layer, including peaks 1–4 from the solvent fractioning, was separated by a Sephadex LH-20 column eluted with 60% methanol because the *K* values of each component are overlapped. Four compounds were observed and compound 2 was purified by recycle-HPLC to improve its purity. In two experiments, the four compounds (1–4) were evaporated and measured, and the weights were 3.2, 5.0, 3.8, and 44.5 mg at 92.3, 98.1, 89.7, and 95.8% purity, respectively, as determined by HPLC (Figure 4). All nine compounds were identified by comparing ^1^H and ^13^C-NMR, EI-MS, and UV to previously reported data [16–25]. The compounds were chlorogenic acid (1), formononetin-7-*O*-*β*-D-glucosyl [1–6] glucoside (2),* trans*-ferulic acid (3), tectoridin (4), piceatannol (5), resveratrol (6), daidzein (7), genistein (8), and formononetin (9) (Figure 5).

*Compound 1.* MALDI-TOF MS *m*/*z* 377.1275 [M + Na]^+^, 191 [M-quinic acid-H]^−^, 179 [M-caffeic acid-H]^−^. UV (MeCN, *λ*_max_ nm) 298, 346. ^1^H-NMR (400 MHz, MeOD): *δ* 7.58 (1H, d, *J* = 15.5 Hz, H-7), 7.06 (1H, d, *J* = 2.0 Hz, H-2′), 6.97 (1H, dd, *J* = 8.0 and 2.0 Hz, H-6′), 6.79 (1H, d, *J* = 8.0 Hz, H-5′), 6.28 (1H, d, *J* = 16.0 Hz, H-8′), 2.05–3.72 (2H, m, H-2, 3 and 6). ^13^C-NMR (400 MHz, MeOD): *δ* 175.8 (C-7), 167.3 (C-9′), 147.2 (C-4′), 145.7 (C-7′), 145.4 (C-3′), 126.4 (C-1′), 122.5 (C-6′), 115.1 (C-8′), 114.1 (C-5′), 113.8 (C-2′), 74.9 (C-1), 72.3 (C-3), 70.1 (C-4), 69.8 (C-5), 36.8 (C-2), 37.6 (C-6).

*Compound 2.* FAB-MS *m*/*z* 609 [M + H]^+^. UV (MeCN, *λ*_max_ nm) 263, 330. ^1^H-NMR (400 MHz, MeOD): *δ* 8.68 (1H, s, H-2), 8.02 (1H, d, *J* = 7.5 Hz, H-5), 7.39 (2H, d, *J* = 8.5 Hz, H-2′ and 6′), 6.98 (1H, dd, *J* = 7.5 and 1.5 Hz, H-6), 7.18 (1H, d, *J* = 3.0 Hz, H-8), 6.85 (2H, d, *J* = 8.7 Hz, H-3′ and 5′), 3.83 (3H, s, H-4′-OCH_3_), 3.43–5.21 (14H, m,* O*-*β*-glucosyl [1–6] glc). ^13^C-NMR (400 MHz, MeOD): *δ* 175.3 (C-4), 161.4 (C-7), 159.8 (C-4′), 157.8 (C-8a), 153.2 (C-2), 130.1 (C-2′), 130.1 (C-6′), 127.4 (C-5), 124.8 (C-1′), 123.5 (C-3), 117.2 (C-4a), 115.7 (C-6), 114.2 (C-3′), 114.2 (C-5′), 109.2 (Glc-1′′), 102.0 (C-8), 100.5 (Glc-1′), 78.1 (Glc-5′′), 76.8 (Glc-3′), 76.8 (Glc-5′), 76.1 (Glc-3′′), 73.8 (Glc-2′′), 73.4 (Glc-2′), 71.5 (Glc-4′′), 70.3 (Glc-4′), 68.6 (Glc-6′), 61.5 (Glc-6′′), 55.8 (4′-OCH_3_).

*Compound 3.* EI-MS *m*/*z* 194 [M]^+^. UV (MeCN, *λ*_max_ nm) 239, 323. ^1^H-NMR (400 MHz, MeOD): *δ* 7.45 (1H, d, *J* = 15.7 Hz, H-7), 7.16 (1H, d, *J* = 2.1 Hz, H-2), 6.79 (1H, d, *J* = 8.3 and 2.1 Hz, H-6), 6.67 (1H, d, *J* = 8.5 Hz, H-5), 6.33 (1H, d, *J* = 15.1 Hz, H-8), 3.83 (3H, s, H-3-OCH_3_). ^13^C-NMR (400 MHz, MeOD): *δ* 171.4 (C-9), 149.1 (C-3), 147.9 (C-4), 146.8 (C-7), 127.6 (C-1), 122.9 (C-6), 119.1 (C-2), 116.4 (C-5), 114.8 (C-8), 56.1 (3-OCH_3_).

*Compound 4.* FAB-MS *m*/*z* 463 [M + H]^+^. UV (MeCN, *λ*_max_ nm) 263, 330. ^1^H-NMR (400 MHz, MeOD): *δ* 8.15 (1H, s, H-2), 7.39 (2H, d, *J* = 8.7 Hz, H-2′ and 6′), 6.88 (1H, s, H-8), 6.85 (2H, d, *J* = 8.7 Hz, H-3′ and 5′), 5.06 (1H, s, Glc-1′′), 3.76 (1H, s, H-6-OCH_3_), 3.51–4.02 (5H, m, Glc-2′′, 3′′, 4′′, 5′′ and 6′′). ^13^C-NMR (400 MHz, MeOD): *δ* 181.2 (C-4), 157.9 (C-4′), 157.1 (C-9), 155.2 (C-2), 153.4 (C-5), 152.9 (C-7), 132.9 (C-6), 130.6 (C-2′), 130.6 (C-6′), 122.5 (C-3), 121.5 (C-1′), 115.6 (C-3′), 115.6 (C-5′), 106.9 (C-10), 100.6 (Glc-1′′), 94.5 (C-8), 77.7 (Glc-5′′), 77.2 (Glc-3′′), 73.6 (Glc-2′′), 70.1 (Glc-4′′), 61.0 (Glc-6′′), 60.8 (C-6-OCH_3_).

*Compound 5.* EI-MS *m*/*z* 243 [M − H]^−^. UV (MeCN, *λ*_max_ nm) 317. ^1^H-NMR (400 MHz, MeOD): *δ* 6.96 (1H, d, *J* = 1.8 Hz, H-2), 6.88 (1H, d, *J* = 16.4 Hz, H-olefinic), 6.82 (1H, dd, *J* = 8.1 and 2.0 Hz, H-6), 6.74 (1H, d, *J* = 16.4 Hz, H-olefinic), 6.73 (1H, d, *J* = 8.0 Hz, H-5), 6.42 (2H, d, *J* = 1.9 Hz, H-2′ and 6′), 6.14 (1H, t, *J* = 2.1 Hz, H-4′). ^13^C-NMR (400 MHz, MeOD): *δ* 158.7 (C-3′), 158.7 (C-5′), 145.3 (C-3), 145.3 (C-4), 141.2 (C-1′), 130.1 (C-1), 127.4 (olefinic), 126.4 (olefinic), 119.6 (C-6), 115.8 (C-5), 115.1 (C-2), 104.9 (C-2′), 104.9 (C-6′), 102.1 (C-4′).

*Compound 6.* EI-MS *m*/*z* 227 [M − H]^−^. UV (MeCN, *λ*_max_ nm) 340. ^1^H-NMR (400 MHz, MeOD): *δ* 7.35 (2H, d, *J* = 8.8 Hz, H-2 and 6), 6.95 (1H, d, *J* = 16.4 Hz, H-olefinic), 6.87 (1H, d, *J* = 16.4 Hz, H-olefinic), 6.75 (2H, d, *J* = 8.8 Hz, H-3 and 5), 6.38 (2H, d, *J* = 2.2 Hz, H-2′ and 6′), 6.27 (1H, t, *J* = 2.2 Hz, H-4′). ^13^C-NMR (400 MHz, MeOD): *δ* 158.2 (C-3′), 158.2 (C-5′), 157.2 (C-4), 140.1 (C-1′), 130.6 (C-2), 130.6 (C-6), 129.1 (C-1), 128.0 (olefinic), 127.4 (olefinic), 115.8 (C-3), 115.8 (C-5), 104.6 (C-2′), 104.6 (C-6′), 101.7 (C-4′).

*Compound 7.* EI-MS *m*/*z* 254 [M]^−^. UV (MeCN, *λ*_max_ nm) 250, 303. ^1^H-NMR (400 MHz, MeOD): *δ* 8.25 (1H, s, H-2), 7.96 (1H, d, *J* = 9.1 Hz, H-5), 7.36 (2H, d, *J* = 8.9 Hz, H-2′ and 6′), 6.90 (1H, dd, *J* = 9.1 and 2.2 Hz, H-6), 6.80 (1H, H-8, d, *J* = 2.0 Hz), 6.72 (2H, d, *J* = 8.9 Hz, H-3′ and 5′). ^13^C-NMR (400 MHz, MeOD): *δ* 178.6 (C-4), 165.0 (C-7), 158.6 (C-2), 158.6 (C-9), 157.7 (C-4′), 138.2 (C-8), 132.1 (C-6), 130.2 (C-2′), 130.2 (C-6′), 125.1 (C-1′), 123.5 (C-3), 118.2 (C-10), 107.0 (C-3′), 107.0 (C-5′), 103.5 (C-5).

*Compound 8.* EI-MS *m*/*z* 270 [M]^+^. UV (MeCN, *λ*_max_ nm) 261, 328. ^1^H-NMR (400 MHz, MeOD): *δ* 8.04 (1H, s, H-2), 7.36 (2H, d, *J* = 8.7 Hz, H-2′ and 6′), 6.84 (2H, d, *J* = 8.4 Hz, H-3′ and 5′), 6.33 (1H, d, *J* = 2.1 Hz, H-8), 6.20 (1H, d, *J* = 2.2 Hz, H-6). ^13^C-NMR (400 MHz, MeOD): *δ* 180.7 (C-4), 166.4 (C-7), 161.8 (C-5), 157.8 (C-9), 157.7 (C-4′), 153.2 (C-2), 130.0 (C-2′), 130.0 (C-6′), 125.1 (C-1′), 122.2 (C-3), 115.8 (C-3′), 115.8 (C-5′), 105.5 (C-10), 98.5 (C-6), 94.1 (C-8).

*Compound 9.* EI-MS *m*/*z* 269 [M + H]^+^. UV (MeCN, *λ*_max_ nm) 253, 300. ^1^H-NMR (400 MHz, MeOD): *δ* 8.16 (1H, s, H-2), 7.96 (1H, d, *J* = 8.7 Hz, H-5), 7.46 (2H, d, *J* = 8.7 Hz, H-2′ and 6′), 6.98 (2H, d, *J* = 8.7 Hz, H-3′ and 5′), 6.93 (1H, dd, *J* = 8.8 and 2.1 Hz, H-6), 6.85 (1H, d, *J* = 1.8 Hz, H-8). ^13^C-NMR (400 MHz, MeOD): *δ* 175.3 (C-4), 160.8 (C-7), 158.6 (C-9), 157.8 (C-4′), 153.1 (C-2), 131.4 (C-2′), 131.4 (C-6′), 128.1 (C-5), 125.3 (C-3), 125.2 (C-10), 125.1 (C-1′), 115.0 (C-6), 114.5 (C-3′), 114.5 (C-5′), 103.4 (C-8), 55.7 (C-4′-OCH_3_).

### 3.5. Activity Assessment of Isolated Compounds

#### 3.5.1. Antioxidant Effect of Isolated Compounds

The antioxidant activities of compounds were confirmed by ABTS assay (Table 3). Compounds 5 and 6 had stilbene structures and showed potent inhibitory activity, with IC_50_ values of 6.73 *μ*M and 11.05 *μ*M as compared to the positive control, Trolox, with an IC_50_ value of 16.83 *μ*M. Compounds 1 and 3, which were phenols, also had higher IC_50_ values compared to Trolox (27.23 *μ*M and 13.51 *μ*M, resp.). Other isolated compounds, 2, 4, and 7–9, were flavonoids that did not have antioxidant activity.

#### 3.5.2. Rat Lens AR Inhibitory Activity of the Isolated Compounds

The ARI were performed to confirm the activities of compounds (Table 3). Compound 1 showed the maximum inhibitory activity of AR with an IC_50_ value of 4.2 *μ*M as compared to the positive control, quercetin, with an IC_50_ value of 10.1 *μ*M. Compound 4 exhibited the second highest activity, with an IC_50_ value of 50.4 *μ*M, and 8, 9, 6, and 7 showed decreasing antioxidant activity, as shown in Table 3. Other isolated compounds, 2, 3, and 5, were flavonoids that did not have RLAR activity.

## 4. Discussion

Diabetic complications including cataracts, neuropathy, nephropathy, and retinopathy caused by several mechanisms can be retarded by inhibiting AR in the polyol pathway and decreasing oxidative stress [3]. In the present study, we investigated the inhibitory effect of MA on RLAR and ABTS assay and could know that it would be useful for treatment of diabetic complications. To rapidly identify screening of active compounds from MA, we used offline HPLC-ABTS assay and microfractionation AR assay using HPLC. The offline HPLC-ABTS assay could identify antioxidants from complex mixtures without isolation process. The peak area of efficacious compounds will decrease in chromatography after adding ABTS; however, other compound peak areas without antioxidant activity are not changed [10]. Microfractionation using HPLC also plays an important role in the search for active compounds from plants, providing rapid access to information concerning both the activity and localization of the activity in complex plant matrices [11]. As shown in Table 3, antioxidant and AR inhibitory activities of compounds well matched the quantitative results of the ABTS-offline HPLC assay and microfractionation using HPLC, respectively. We believe that ABTS-offline HPLC assay and microfractionation using HPLC can be very efficient and fast for screening active compounds from complex mixtures, particularly nature products.

Active components from MA extract were separated by HSCCC, which was widely applied as a convenient and efficient technique. For HSCCC separation, the solvent system is the most important step. Commonly, three factors are considered for two-phase solvent systems. First, to assure safety retention of the stationary phase, the settling time of the solvent system should be within 30 s. The second factor is that the partition coefficient (*K*) of the target compounds has to be within the range 0.5 ≤ *K* ≤ 2.5 for efficient separation. Lastly, the separation factor between the components (*α* = *K*2/*K*1, *K*2 > *K*1) should be greater than 1.5 [26, 27]. In this study, the *K* values of the 5 compounds were determined by HPLC, as described in Materials and Methods. The measured *K* values of each compound are summarized in Table 2. Based on the criteria for *K* values, one system was selected with n-hexane-ethyl acetate-methanol-water (2.5 : 7.5 : 5 : 5, v/v) and five compounds (5–9) isolated by its system were evaporated to yield 170.1, 94.6, 8.2, 10.4, and 5.5 mg.

The structure activity relationship (SAR) of active compounds was investigated using the antioxidant and RLAR assay. Stilbenes structures, compounds 5 and 6, showed the highest antioxidant activity, and compound 5 had more antioxidant activity than compound 6 because it has four hydroxyl groups, including a catechol structure in the B ring [28]. The phenolic compounds showed the second highest antioxidant activity and as compared to compounds 1 and 3, compound 3 appears to have more high antioxidative efficiency caused by their methoxy group [29]. In SAR of RLAR assay, active compounds were divided into three groups as phenolic acids, stilbenes, and flavonoids, as shown in Figure 3. Compound 1, a phenolic acid, is commonly found in plants and is a known antioxidant, metal chelator, and AR inhibitor in vitro [30]. Compound 3, with a similar structure, showed lower inhibition of AR because it has a methyl group instead of quinic acid. Flavonoid compounds also showed high inhibition of RLAR. Compound 4 exhibited effective activity because of a hydroxyl group and a methoxyl group in ring A, which is associated with AR inhibitory activity [31]. The activity of compounds 7 and 8 was attributed to the free hydroxyl group at C-7, which is a significant component for the inhibitory efficacy of AR. First, these have the ability to form a hydrogen bond with amino acids Tyr48 and His110 in an enzyme's active site [32]. Furthermore, 2-phenyl substitution was found to be a suitable hydrophobic pocket of the enzyme lined with amino acids Trp111 and Leu300 due to its aromatic fragment and lipophilic nature as well as its specific spatial conformation [33]. Finally, the 40-hydroxyl group seems to play an important role in the ARI activity of these compounds, as it can form a bond to amino acid Thr113 [34]. Compound 8 has previously been reported to have an inhibitory effect on AR activity and increase GSH levels, which may help to prevent xylose-induced opacity of diabetic lenses [35]. Compound 8 had better inhibition than 7 because it has a hydroxyl group in ring A, as mentioned above. Another flavonoid compound, compound 9, also exhibited inhibitory activity against AR owing to the substitution of a hydroxyl group with a methoxy group at C-4′ [36]. When we compared compounds 6 and 5, which contain stilbenes, the activity of 6 without the hydroxyl group at C-3′ was one hundred times higher than that of 5. Compound 6 is already known as an antioxidant and effectively reduces blood sugar in streptozotocin-induced diabetic rats and normalizes renal dysfunction in diabetic rats [37]. Compound 6 increased superoxide dismutase, catalase, glutathione peroxidase, glutathione-S-transferase, and glutathione reductase activities and vitamins C and E and reduced glutathione levels, with a significant decline in lipid peroxide, hydroperoxide, and protein carbonyl levels in diabetic kidneys [38].

## 5. Conclusion

The ABTS-HPLC offline radical scavenging analysis method and HPLC microfractionation system are the rapid determination methods of active components in MA. Based on the rapid determination methods, HSCCC was successfully applied to separate and purify compounds using hexane-ethyl acetate-methanol-water (2.5 : 7.5 : 5 : 5, v/v) as the solvent system and four compounds were isolated using the Sephadex LH-20 column chromatography. In addition, the RLAR and ABTS radical scavenging inhibitory activities of MA and its constituents were investigated. MA and its constituents showed high inhibitory activities regarding RLAR and ABTS. Among them, chlorogenic acid and piceatannol were identified as potential antioxidants and RLAR inhibitors. Therefore, our results suggested that HSCCC based on ABTS-HPLC offline analysis method and HPLC microfractionation system are a powerful and fast technique for determining, separating, and purifying active compounds from natural sources and MA can be a potent functional food ingredient as AR and protective of oxidative stress.

## Figures and Tables

**Table 1:** Effects of *Maackia amurensis *fractions in terms of antioxidation and inhibition of aldose reductase in rat lens.

Test sample	Antioxidant		RLAR inhibition
	IC_50_ (*μ*g/mL)^a^	TEAC^b^	IC_50_ (*μ*g/mL)
Crude	12.03 ± 0.02	0.72	35.6 ± 3.82
*n*-Hex fraction	134.02 ± 0.78	0.07	>100
CH_2_Cl_2_ fraction	141.13 ± 0.78	0.06	>100
EtOAc fraction	4.92 ± 0.02	1.77	11.3 ± 4.31
*n*-BuOH fraction	96.48 ± 0.45	0.09	>100
H_2_O fraction	282.62 ± 0.38	0.03	>100
Trolox^c^	8.72 ± 0.01	1.00	—
Quercetin^d^	—	—	3.30 ± 3.20

^a^The IC_50_ value was defined as the concentration of the 50% inhibition.

^b^Trolox equivalent antioxidant capacity.

^c^Trolox was used as positive control for ABTS assay.

^d^Quercetin was used as positive control for RLAR inhibitory activity measurement.

**Table 2:** The partition coefficients (*K*) of the target compounds in several solvent systems.

Types of solvent system	Ratio (v/v)	Settling time (s)	*K* values				
			Peak 5	Peak 6	Peak 7	Peak 8	Peak 9
*n*-Heptane-ethyl acetate-methanol-water	1 : 6 : 1 : 6	9	—	9.56	—	—	—
*n*-Hexane-ethyl acetate-methanol-water	1 : 3 : 1 : 3	13	—	—	—	35.34	—
*n*-Hexane-ethyl acetate-methanol-water	3 : 7 : 4.5 : 5.5	25	0.34	0.59	—	2.52	0.40
*n*-Hexane-ethyl acetate-methanol-water	2.5 : 7.5 : 5 : 5	23	0.37	0.57	1.01	2.04	0.33
*n*-Hexane-ethyl acetate-methanol-water	4 : 6 : 3.5 : 5.5	30	0.37	0.90	0.69	3.73	0.39
*n*-Hexane-ethyl acetate-methanol-water	4 : 6 : 2.5 : 7.5	28	3.91	4.62	—	15.01	3.39

Peak 5, piceatannol; peak 6, resveratrol; peak 7, daidzein; peak 8, genistein; peak 9, formononetin.

**Table 3:** Antioxidant and aldose reductase inhibitory effects in rat lens of nine compounds isolated from the ethyl acetate fraction of *Maackia amurensis*.

Isolated compounds	Antioxidant		RLAR inhibition	
	Quantitative reduction (%)^a^	IC_50_ (*μ*M)^b^	Inhibition (%)^c^	IC_50_ (*μ*M)
Chlorogenic acid (1)	17.09	27.23 ± 0.17	51.02	4.2 ± 3.20
Formononetin-7-*O*-*β-*D*-*glucosyl [1–6] glucoside (2)	1.04	>500	2.32	>500
*trans*-Ferulic acid (3)	20.06	13.51 ± 0.04	5.21	>500
Tectoridin (4)	8.43	>500	43.37	50.4 ± 2.17
Piceatannol (5)	34.68	6.73 ± 0.04	5.47	>500
Resveratrol (6)	29.96	11.05 ± 0.04	29.31	117.6 ± 3.12
Daidzein (7)	0.48	>500	28.14	151.9 ± 2.91
Genistein (8)	2.24	>500	38.54	57.1 ± 3.23
Formononetin (9)	0.69	>500	30.11	69.2 ± 2.57
Trolox^d^	—	16.83 ± 0.06	—	—
Quercetin^e^	—	—	—	10.1 ± 2.18

^a^Quantitative reduction was defined as the amount of decrease in ABTS-offline HPLC.

^b^The IC_50_ value was defined as the concentration of the 50% inhibition.

^c^Inhibition was defined as the amount of efficacy in HPLC microfractionation.

^d^Trolox was used as positive control for ABTS assay.

^e^Quercetin was used as positive control for RLAR inhibitory activity measurement.

## Abbreviations

ABTS: 2,2′-Azino-bis(3-ethylbenzothiazline-6-sulfonic acid) diammonium salt
AR: Aldose reductase
*n*-BuOH: * n*-Butanol
CH_2_Cl_2_: Methylene chloride
DMSO: Dimethyl sulfoxide
EtOAc: Ethyl acetate
*n*-Hex: * n*-Hexane
H_2_O: Distilled water
HSCCC: High-speed countercurrent chromatography
*K*: Partition coefficient
MA: * Maackia amurensis*
NADPH: Nicotinamide adenine dinucleotide phosphate
NADH: Nicotinamide adenine dinucleotide
RLAR: Rat lens aldose reductase
SAR: Structure activity relationship
TEAC: Evaluation of trolox equivalent antioxidant capacity
Trolox: 6-Hydroxy-2,5,7,8-tetrame-thylchroman-2-carboxylic acid.
